# Cavitation Damage Prediction in Mercury Target for Pulsed Spallation Neutron Source Using Monte Carlo Simulation

**DOI:** 10.3390/ma16175830

**Published:** 2023-08-25

**Authors:** Takashi Wakui, Yoichi Takagishi, Masatoshi Futakawa

**Affiliations:** 1 J-PARC Center, Japan Atomic Energy Agency, Ibaraki 319-1195, Japan; futakawa.masatoshi@jaea.go.jp; 2 Kobelco Research Institute Inc., Kobe 651-2271, Japan; takagishi.yoichi@kki.kobelco.com

**Keywords:** mercury target, cavitation damage, Monte Carlo simulation, Gaussian distribution, delta function, Weibull distribution, Bayesian optimization

## Abstract

Cavitation damage on a mercury target vessel for a pulsed spallation neutron source is induced by a proton beam injection in mercury. Cavitation damage is one of factors affecting the allowable beam power and the life time of a mercury target vessel. The prediction method of the cavitation damage using Monte Carlo simulations was proposed taking into account the uncertainties of the core position of cavitation bubbles and impact pressure distributions. The distribution of impact pressure attributed to individual cavitation bubble collapsing was assumed to be Gaussian distribution and the probability distribution of the maximum value of impact pressures was assumed to be three kinds of distributions: the delta function and Gaussian and Weibull distributions. Two parameters in equations describing the distribution of impact pressure were estimated using Bayesian optimization by comparing the distribution of the cavitation damage obtained from the experiment with the distribution of the accumulated plastic strain obtained from the simulation. Regardless of the distribution type, the estimated maximum impact pressure was 1.2–2.9 GPa and existed in the range of values predicted by the ratio of the diameter and depth of the pit. The estimated dispersion of the impact pressure distribution was 1.0–1.7 μm and corresponded to the diameter of major pits. In the distribution of the pits described by the accumulated plastic strain, which was assumed in three cases, the delta function and Gaussian and Weibull distributions, the Weibull distribution agreed well with the experimental results, particularly including relatively large pit size. Furthermore, the Weibull distribution reproduced the depth profile, i.e., pit shape, better than that using the delta function or Gaussian distribution. It can be said that the cavitation erosion phenomenon is predictable by adopting the Weibull distribution. This prediction method is expected to be applied to predict the cavitation damage in fluid equipment such as pumps and fluid parts.

## 1. Introduction

Neutron measurements are expected to promote the progress of innovative science in the fields of materials science, chemistry, physics and biology [1,2,3,4]. Technologies and large-scale experimental facilities to supply high-intensity neutrons have been constructed globally [5]. Innovative neutrons have the great advantage of being able to detect light elements such as hydrogen. Furthermore, the absorption cross section for neutrons is much smaller than an X-ray, and therefore neutrons easily transmit through materials. Neutrons are expected to contribute the development of various elemental technologies, particularly for realizing a carbon-neutral society, such as through the use of fuel cells [6].

High-intensity proton beams are injected into the target materials to generate high-intensity neutrons to perform high-precision neutron measurements in a short period of time. The temperature of the target material rises rapidly due to the spallation reaction of the target materials, and the amount of temperature rise increases as the power of the proton beam increases. A liquid heavy metal, mercury, having functions of a cooling medium and a spallation material, was applied in order to efficiently remove a large amount of heat from the target and generates high-intensity neutrons in J-PARC (Japan Proton Accelerator Research Complex) and SNS (Spallation Neutron Source) [7,8]. When high-intensity proton beams are injected into the mercury in a mercury vessel, pressure waves in mercury induce the growth, shrinkage and collapse of cavitation bubbles scatted in the mercury. And then cavitation damage is added to the inner surface of the mercury vessel due to the impact pressure caused by the localized pressure generated when the cavitation bubbles collapse near the interface between the solid wall and mercury. From the viewpoint of integrity and durability under high power operation, it is essential to appropriately predict the degree of cavitation damage, which is associated with the localized impact due to the collapsing. Tests on cavitation damage with localized impact in water have been conducted using various test techniques. The cavitation damage with localized impacts caused by laser- or hydrodynamic-spark-induced bubble collapse was investigated [9,10,11,12] and the impact force was measured using a force sensor based on optical beam deflection at different laser energies [9]. The cavitation damage caused by ultrasonic testing or pressure bar was also investigated [13,14,15,16,17]. In order to capture the implosion mechanism of cavitation bubbles generated by an ultrasonic transducer, bubble behavior near the boundary was identified using a high-speed camera and the damaged surface was examined using high-precision 3D optical interferometer techniques. The impact pressure based on experimental results was linearly proportional to the deformed area of the pits [14]. The impact pressure estimated from the shape of individual cavitation pits caused by the high-velocity flow was investigated using a numerical calculation and a finite element method assuming an indentation test [18,19,20,21]. As for the mercury targets, cavitation damage tests were conducted using mercury to investigate the cavitation damage evolution on the surface in contact with mercury [22,23,24,25,26,27]. The cavitation damage on the inner surface of the used mercury targets was investigated [28,29,30,31,32].

**Figure 1:**
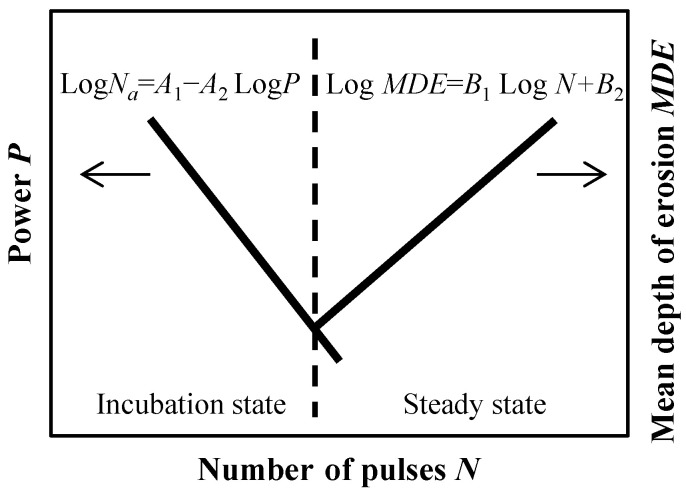
The conceptual diagram to evaluate the life time of the mercury target vessel.

**Figure 2:**
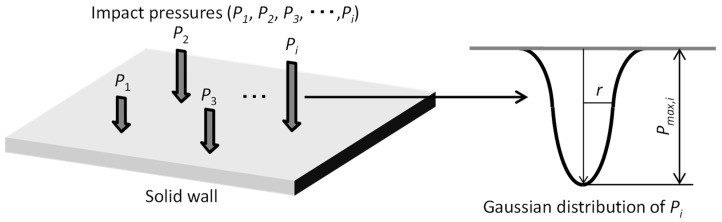
Impact pressures are applied on the solid wall *i* times. The distribution of each impact pressure, *P_i_*, is assumed to be Gaussian distribution with the maximum value, *P_max_*_,*i*_, and the dispersion, *r*.

Figure 1 shows a conceptual diagram to estimate the life time and allowable number of pulses of the mercury target vessel from the view point of the cavitation damage taking the beam power into account. The cavitation damage might be divided into two states: the incubation state and the steady state. In the incubation state, the mass loss is hardly induced by the cavitation damage and the allowable number of pulses, and *N_a_* is reduced with the increase in power, *P*. Based on the results of the cavitation damage test, the following empirical formula for the relationship between the allowable number and the power was proposed [23].

(1) $$\mathrm{Log}N_{a}=A_{1}-A_{2}\cdot \log P,$$

where *A*_1_ and *A*_2_ are constant in terms of materials. On the other hand, the mass loss due to the cavitation damage increases when the number of pulses exceeds the allowable number of pulses. Based on the results of the cavitation damage test, the following empirical formula for the relationship between the mean depth of erosion, *MDE*, and the number of pulses was proposed [23]:

(2) $$\mathrm{Log}MDE=B_{1}\cdot \log N+B_{2},$$

where *B*_1_ and *B*_2_ are constant in terms of materials. However, the impact pressure due to the collapse of the cavitation bubble depends on the sizes of the cavitation bubbles, distances of the cavitation bubbles to a solid wall, pressure fluctuations around the cavitation bubbles, etc. It is necessary to conduct a probabilistic discussion taking their frequency of occurrence into account. Therefore, a method for predicting the damage growth using Monte Carlo simulation was proposed considering the impact pressure distribution and the scatter in the position of cavitation bubbles [33]. In the method, the distribution of impact pressure *P_i_* with the maximum value *P_max_*_,*i*_ and the dispersion *r* due to each bubble collapse was assumed to be Gaussian distribution, as shown in Figure 2. Furthermore, the probability density function of the maximum values *P_max_*_,*i*_ in the impact pressure distribution was also assumed to be Gaussian distribution. Positions of the impact pressure were randomly placed on the plane. It has been reported that the simulation results roughly reproduced experimental results [33].

In this study, the probability density function of the maximum impact pressure was focused on, and the damage evaluation was conducted using three types of probability density functions: the delta function and Gaussian and Weibull distributions. Parameters in the equation representing the impact pressure distribution were determined. Distributions of the pit diameter, the fraction of the damaged area and the accumulated plastic strain in the depth direction were evaluated and experimental results were compared. And then the effect of the probability density function of the maximum impact pressure on evaluation results of the cavitation damage was investigated.

## 2. Experiments

Cavitation damage tests were conducted using an MIMTM (electromagnetic IMpact Testing Machine, Shinken co., Ltd, Hachiouji, Japan) [22], which was developed to investigate the growth behavior of the cavitation damage on the inner surface of the mercury target vessel due to proton beam injections. The impulsive pressure was imposed to the mercury by controlling the electric power to electromagnetic coils in the MIMTM. The morphology of cavitation damage produced by the MIMTM with the power of 560 W is sufficiently equivalent to that in the on-beam tests with MW-class proton beams. The polished surface of the disk specimen with a diameter of 100 mm and a thickness of 1 mm was divided into 6 regions and each region was individually exposed to mercury for up to 10 million impact cycles. The material was 316 austenitic stainless steel.

The surface shape of the damaged region was measured three-dimensionally by using a laser microscope (KEYENCE, VK-9500, Keyence Corporation, Osaka, Japan). The measurement area in the damaged region was more than 10 mm^2^. Measurement accuracies in the in-plane direction and the depth direction were 0.275 μm and 0.01 μm, respectively. The estimation of the pit diameter and counting of the number of pits were conducted by using an image analyzer (KEYENCE, VK Analyzer Plus ver.2.4.0.0) [34].

## 3. Numerical Analysis

### 3.1. Flow of Analysis

**Figure 3:**
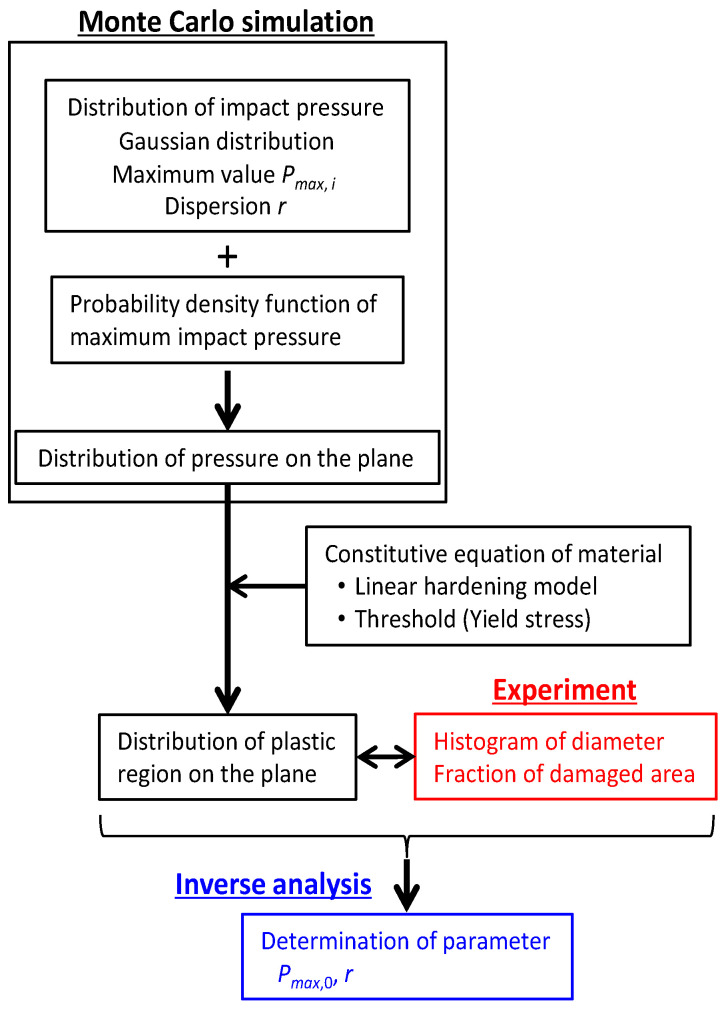
The flow of the analysis to determine parameters using the inverse analysis (blue) by comparing between results of the Monte Carlo simulation (black) and experiment (red).

**Figure 4:**
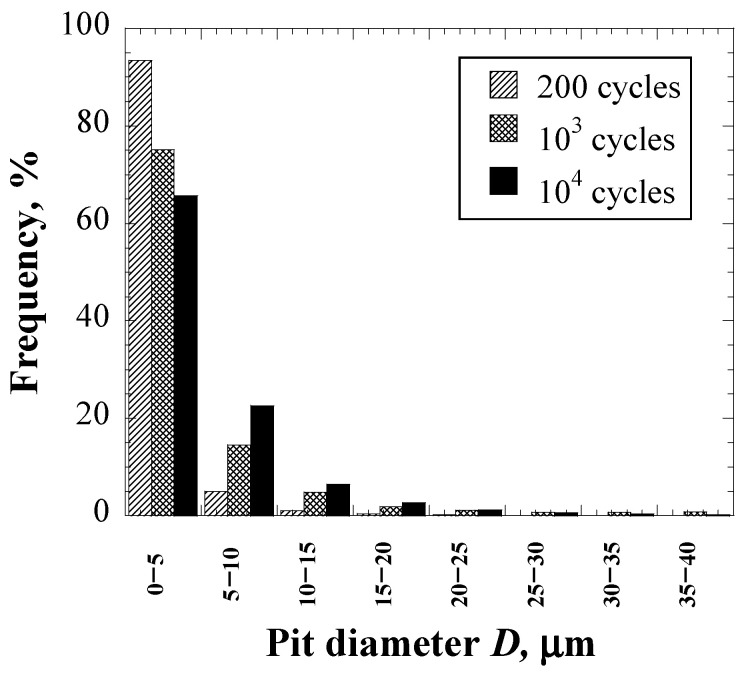
The histogram of the pit diameter.

**Figure 5:**
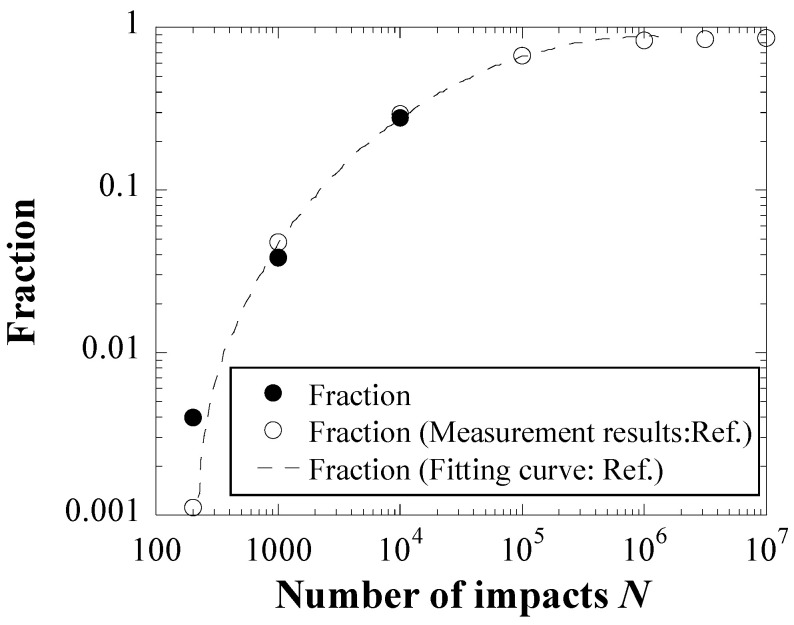
Relationships between the fraction of the damaged area and the number of cycles.

Figure 3 shows the flow of analysis to determine parameters that can reproduce experimental results. Parameters to determine were the reference value of the maximum impact pressure *P_max_*_,0_ described in Section 3.2 and the dispersion *r* of the impact pressure distribution. The distribution of the impact pressure was assumed to be Gaussian distribution with the maximum impact pressure *P_max_*_,_*_i_* and the dispersion *r*. Furthermore, the maximum impact pressure was defined using probability density functions. Monte Carlo simulations were conducted by repeating the impact pressure loading taking the randomness of impact points on the plane and the distribution of the localized impact pressure into account, and then the distribution of pressure applied on the plane was obtained. In order to compare simulation results with the experimental results, the distribution of the plastic region on the plane was evaluated from the distribution of the pressure applied to the plate. A linear work hardening model represented by Equation (3) was applied as the relationship between the plastic stress *σ_p_* and the plastic strain *ε_p_* [35].

(3) $$\sigma_{p}=\sigma_{y}+C\cdot \varepsilon_{p},$$

where *σ_y_* is the yield stress and *C* is the work hardening coefficient. Values of *σ_y_* and *C* are 203 GPa and 958 MPa, respectively [18]. The yield stress was used as the threshold for determining the plastic region from the impact pressure distribution. In the inverse analysis with Bayesian optimization, the parameters including the reference value of the maximum impact pressure and the dispersion of the impact pressure distribution were determined by minimizing the difference between simulation and experimental results. The experimental results for comparison with the simulation results are shown in Figure 4 and Figure 5. The diameter and area of each pit were measured from images of the damaged surface, and then the histogram of pit diameters *D* and the fraction of damaged areas were obtained. The proportion of pits with *D* < 5 μm was high, and the proportion decreased as the pits became larger. As the number of cycles increased, the proportion of pits with *D* < 5 μm gradually decreased and the proportion of pits with *D* > 5 μm gradually increased. The fraction of the damaged area gradually increased as the number of cycles increased and approached 1, like the fitting curve based on the experimental result [28].

### 3.2. Modeling Scatter of Cavitation Damage

**Figure 6:**
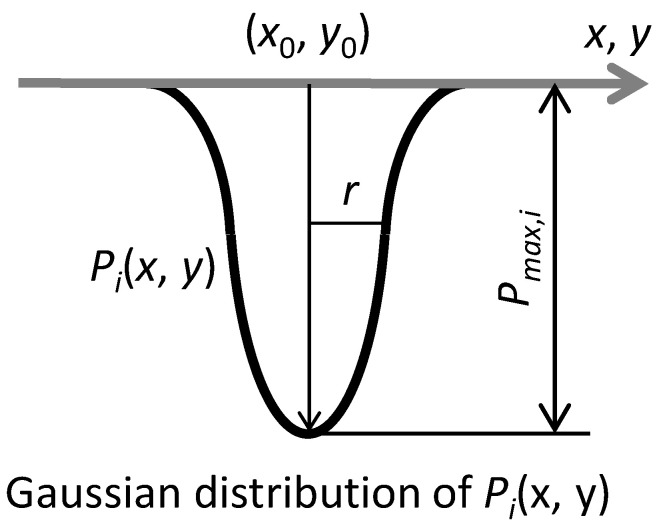
The Gaussian distribution of the impact pressure at the location (*x*, *y*).

The growth behavior of the cavitation damage against the number of cycles was simulated using the Monte Carlo method. The distribution of the impact pressure was defined by Gaussian distribution and the plate suffered the impact pressure at a random position for each impact cycle. When the center point of the Gaussian distribution and the maximum impact pressure were defined as (*x*_0_, *y*_0_) and *P_max_*_,*i*_, the impact pressure distribution *P_i_*(*x*, *y*) at the location (*x*, *y*) as shown in Figure 6 was represented in Equation (4).

(4) $$P_{i}\left(x,y\right)=P_{max,i}exp\left[-\frac{{\left(x-x_{0}\right)}^{2}+{\left(y-y_{0}\right)}^{2}}{r^{2}}\right]\;,$$

where *r* is the dispersion of the impact pressure distribution. Furthermore, it was assumed that the maximum impact pressure *P_max_*_,*i*_ followed the probability density functions.

(5) $$P_{max,i}=f\cdot P_{max,0},\;\;$$

where *P_max_*_,0_ was the reference value of the maximum impact pressure. The probability density functions *f* were assumed as the following: the delta function and Gaussian and Weibull distributions, shown in Equations (6)–(8).

**Figure 7:**
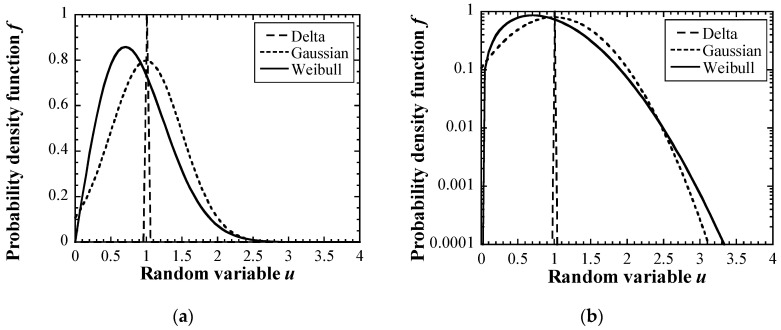
Probability density functions of the delta function and Gaussian and Weibull distributions: (**a**) normal plot, (**b**) semi-log plot.

(6) Delta: f=1,

(7) $$\mathrm{Gaussian}:\;f=\frac{1}{\sqrt{2\pi \sigma^{2}}}exp\left[-\frac{{\left(u-1\right)}^{2}}{2\sigma^{2}}\right],$$

(8) $$\mathrm{Weibull}:\;f=\frac{k}{\eta }{\left(\frac{u}{\eta }\right)}^{k-1}exp\left[-{\left(\frac{u}{\eta }\right)}^{k}\right],$$

where *σ*^2^ is the dispersion of the Gaussian distribution and the value of *σ* was defined as a half of *P_max_*_,*i*_ in this study; *u* is the random variable; and *k* and *η* are the shape parameter and the scale parameter, and these values are 2 and 1, respectively, in this study. Figure 7 shows the probability density functions with (a) normal plots and (b) semi-log (Y-axis log scale) plots, respectively. The mode of the Weibull distribution is lower than those of the delta function and the Gaussian distribution. Note that the probability density of *u* larger than 2.5 in the Weibull distribution is higher than that in the Gaussian distribution, as described in Figure 7b. This indicates that the probability of an extremely large impact is likely to occur when the *P_max_*_,*i*_ follows the Weibull distribution.

### 3.3. Monte Carlo Simulation

**Figure 8:**
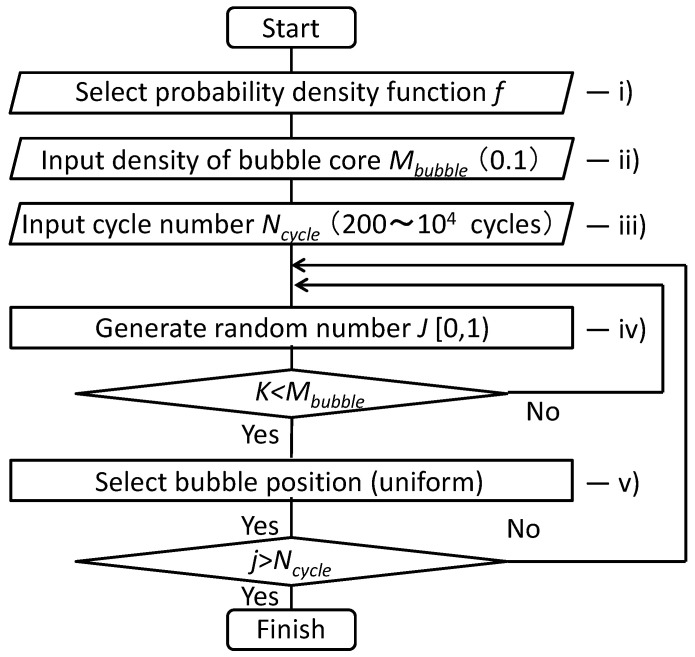
The flow diagram of the simulation using the Monte Carlo method.

The flow diagram of the simulation using the Monte Carlo method [36,37,38] is shown in Figure 8.

(i) The probability density function of the maximum impact pressure was selected from Equations (6)–(8).
(ii) The density of the bubble core *M_bubble_* was inputted to the model. It was assumed that 0.1 bubbles per cycle affect the simulation area measuring 200 μm × 200 μm. This frequency was calculated from the measured bubble density [24].
(iii) The number of impact cycles *N_cycle_* was inputted to the model from 200 to 10^4^.
(iv) The random number between 0 and 1 was generated, and then (iv) was repeated while increasing the number of the iteration *j* by 1 until *K* was less than *M_bubble_*.
(v) The bubble position was selected using a uniform random number in the area measuring 200 μm × 200 μm, and then (iv) and (v) were repeated while increasing the number of the iteration *j* by 1 until *j* was larger than *N_cycle_*.

### 3.4. Inverse Analysis

The expected value *P_max_*_,0_ in each distribution of the maximum impact pressure and the dispersion *r* of the impact pressure distribution were determined so that the simulation results corresponded to the experimental results. Comparison targets were the histogram of the pit diameter and the fraction of the damaged area on the surface of the specimen-loaded impacts of 200, 10^3^ and 10^4^ cycles. The time-integrated value of the difference between the simulation and experimental results was defined as the evaluation function as shown Equation (9).

(9) $$F=\frac{1}{N}\sum_{i}\sqrt{{\left(h_{i,exp}-h_{i,mc}\right)}^{2}}+\sqrt{{\left(\theta_{i,exp}-\theta_{i,mc}\right)}^{2}}\;\;,$$

where *h_i_* and *θ_i_* are differences in the histogram of the pit diameter and the fraction of the damaged area. The subscripts *exp* and *mc* indicate the experiment and the Monte Carlo simulation. Bayesian optimization [39,40,41] was adopted as a search of the expected value and the dispersion (standard deviation) of the pressure distribution which minimized the evaluation function. A Matern3/2 kernel was used as the covariance function, as shown in Equations (10) and (11).

(10) $$Fk\left(x-x\prime \right)=\left(1+\frac{\sqrt{3}r}{l}\right)exp\left(-\frac{\sqrt{3}r}{l}\right)\;\;,$$

(11) r=∥x−x′∥,

where *l* is the control parameter. The expected improvement was used in the acquisition function. The calculation of Bayesian optimization was conducted using MATLAB2021a Statistics and Machine Learning Toolbox [42].

## 4. Results

### 4.1. Inverse Analysis on Expected Value P_max,0_ and Dispersion r

**Figure 9:**
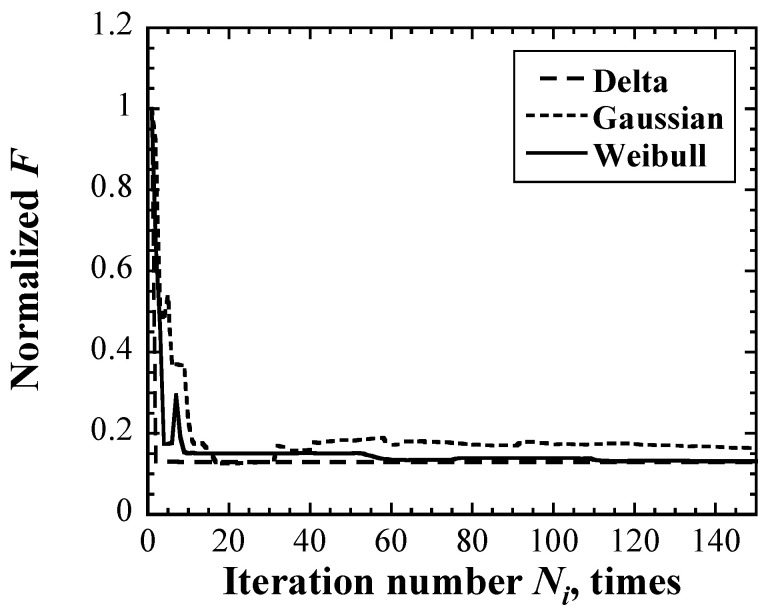
Convergence plots of each probability density function.

The expected values of *P_max_*_,0_ in the delta function and Gaussian and Weibull distributions of the maximum impact pressure and the dispersions *r* of the distribution of the impact pressure were determined using Bayesian optimization combined with Monte Carlo simulations, as described in Section 3. The convergence plot of each probability density function up to 150 iterations is shown in Figure 9. The error in each calculation decreased with the iterations. It decreased significantly by 15 iterations and almost converged within 40 iterations. In this study, the optimization calculations were stopped at 50 iterations and then the *P_max_*_,0_ and *r* were adopted as the optimized values.

**Figure 10:**
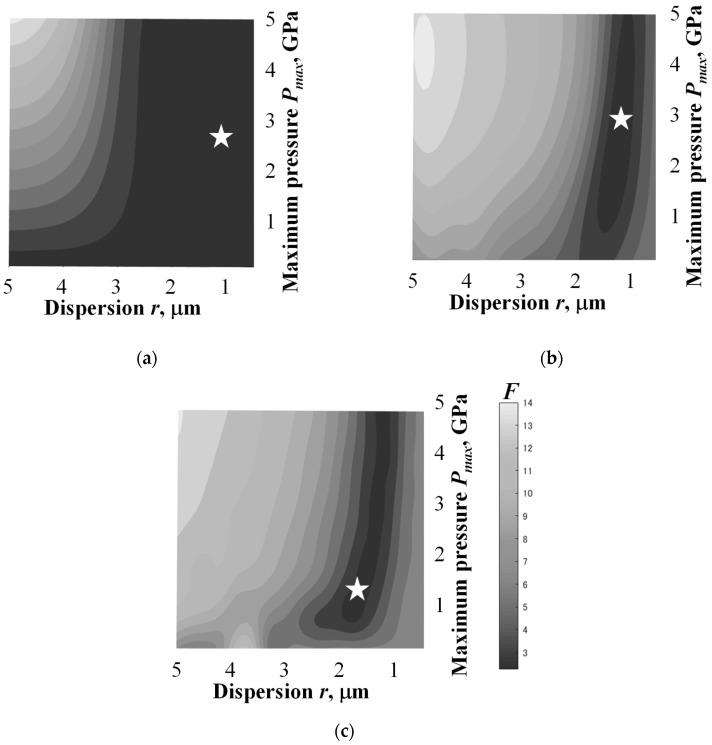
Maps of the evaluation function obtained by using (**a**) the delta function, (**b**) Gaussian distribution and (**c**) Weibull distribution. The asterisk in each figure marks the optimized point with the smallest F.

Figure 10 illustrates the map of the evaluation function *F* in each probability density function of the maximum impact pressure in the range of 0.5 GPa to 5 GPa and the dispersion in the range of 0.5 μm to 5 μm. A smaller *F* and darker gray mean a smaller difference between the simulation and experimental results. In any probability density function, the value of *F* tends to be large when the maximum pressure and dispersion of the impact pressure distribution are large. In the case of the delta function, the change in *F* is small in the range below 2.5 μm of *r*. On the other hand, *F* increases in the range below 1.5 μm with the decrease in *r*. This indicates that the optimal combination of *P_max_*_,0_ and *r* exists in the search range of the system. The asterisk in each figure marks the optimized point with the smallest *F*. The optimized *P_max_*_,0_ in the cases of the delta function and Gaussian and Weibull distributions were 2.9, 2.7 and 1.2 GPa, respectively, and these values were within the range of the experimental results of 0.5–5 GPa. The optimized r in the cases of the delta function and the Gaussian and Weibull distributions were 1.0, 1.2 and 1.7 μm, respectively, and these values were within the range of the major pit diameter of 0–5 μm. It is considered that the result obtained using the Weibull distribution has a smaller *P_max_*_,0_ and a larger *r* than the results obtained using the other functions, because the dispersion of the probability density function widens in the order of the Weibull distribution, the Gaussian distribution and the delta function.

### 4.2. Comparison with Experimental Results

**Figure 11:**
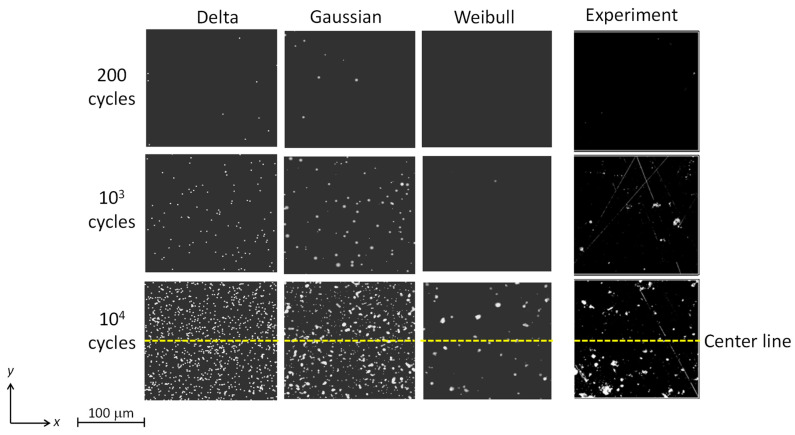
Distributions of accumulated plastic strain obtained by using the delta function and the Gaussian and Weibull distributions and photographs of damaged surface specimen.

Using the values of *P_max_*_,0_ and the dispersion *r* determined in Section 4.1, Monte Carlo simulations (forward simulations) of cavitation damages were conducted until 10^4^ cycles. In the simulation, plastic strains calculated from the impact pressure distribution were superimposed with increasing the number of cycles. In order to compare the simulation results with the experimental results, accumulated plastic strains including individual plastic strains were described as pits in the simulation. Figure 11 illustrates the snapshots of the simulated accumulated plastic strain and the optical microscope images on the damaged surface of the specimen at 200, 10^3^ and 10^4^ cycles, respectively. In the case that the delta function was adopted as the probability density function, many small pits with the same size (accumulated plastic strain regions) were observed in each cycle number. The number of pits increased as the number of cycles increased. The snapshot of the Gaussian function case shows that the number of pits increased as the number of cycles increased, as did the result obtained by using the delta function. On the other hand, unlike the results obtained by using the delta function, the pits had a larger variation in sizes and overlapped each other at 10^4^ cycles in particular. In the case of the Weibull distribution, the number of pits was remarkably small even at 10^4^ cycles, and the pit size was obviously larger than the other two cases. In the simulation results at 10^4^ cycles, the result obtained by using the Weibull distribution appeared to be most similar to the experimental result.

**Figure 12:**
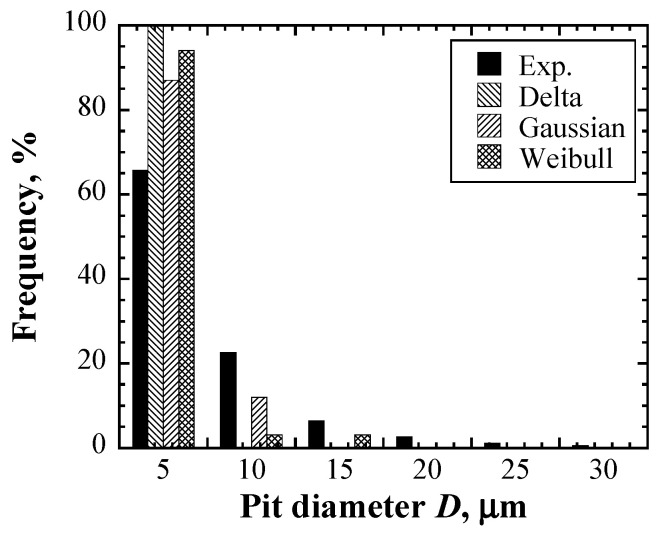
Comparisons of the histogram of the pit diameter between experiment results and Monte Carlo simulations at 10^4^ cycles.

In order to compare the simulation and experimental results in detail, the histograms and faction of the damaged area were confirmed. The histograms of the simulated and actually measured pit diameter *D* at 10^4^ cycles are described in Figure 12. The number of pits with *D* < 5 μm is largest in all the simulated and experimental results. In the range of 5–10 μm, no pits were observed in the delta function case, whereas some pits exist in the Gaussian and Weibull distribution cases as well as in the experiment results, and it could be seen that the frequency of the Gaussian case in this range was close to the frequency of the actual measurement. The pits in both the delta function and the Gaussian distribution cases in the range of 10–15 μm did not exist, although some pits were observed in the Weibull case and the experiment. These results suggest that the pits’ diameter distribution in the Weibull distribution agreed well with the experimental results, particularly including relatively large pit size.

**Figure 13:**
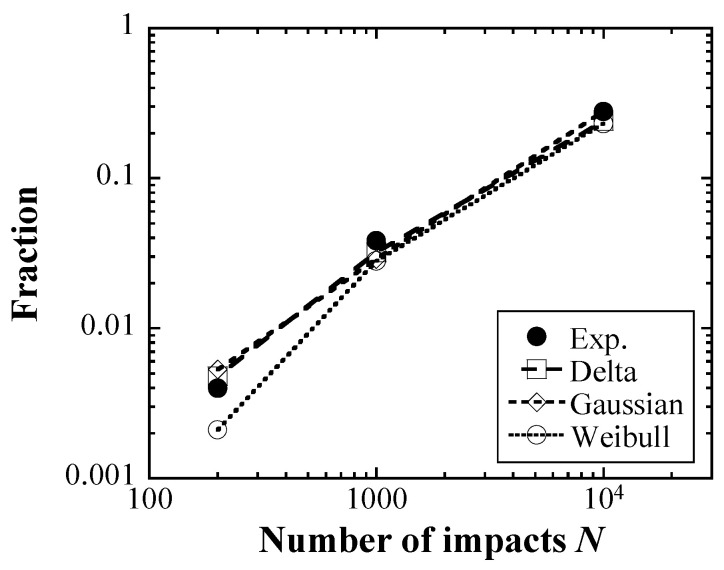
Comparisons of the fraction of the damaged area in each cycle number between experiment result and Monte Carlo simulations.

A comparison of the fraction of the damaged area in each cycle number between the experiment and the Monte Carlo simulations is shown in Figure 13. The fractions of the damaged area evaluated using the delta function and the Gaussian distribution increased with cycle number, as did the fraction obtained in the experiment, whereas the fractions evaluated using the Weibull distribution had slightly lower values. In particular, the fraction obtained by using the Weibull distribution in the case of 200 cycles was about half of the experimental result. This indicates that the followability and predictivity of the damaged area by the Weibull distribution are relatively low, in contrast to those of the pit diameter.

## 5. Discussions

### 5.1. Local Deformation of Pit

**Figure 14:**
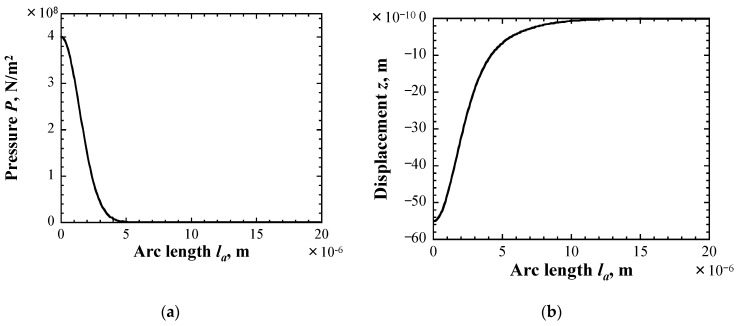
Distributions of (**a**) pressure and (**b**) displacement.

As for the local deformation, the linear correlation between the pressure distribution and the deformation was assumed in the Monte Carlo simulation, as described in Section 3. However, this simple assumption is not always obvious, particularly for the case of large deformation. For this reason, the numerical analysis using the finite element code COMSOL Multiphysics™ ver.5.4 [43] was conducted to investigate the deformation of the surface when the impact pressure distribution shown in Equation (4) was applied to the surface of the material with the material property shown in Equation (3). The distribution of the impact pressure used in the analysis as an input condition and the displacement obtained from the analysis are shown in Figure 14. The displacement roughly corresponded to the distribution of the impact pressure, suggesting that the above simple assumption could be valid in the range of the impact pressure in this study.

### 5.2. Physical Meanings of Probability Distribution Functions

**Figure 15:**
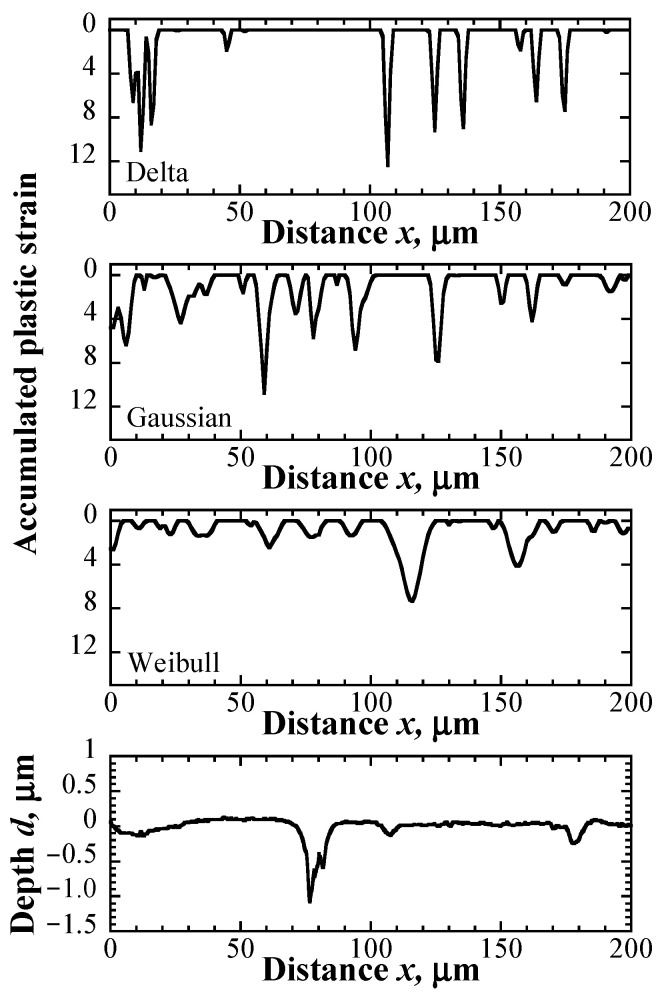
Distributions of accumulated plastic strain in the depth direction and the depth profile of the damaged specimen after 10^4^ cycles.

Figure 15 illustrates the result of the line analyses on the depth profile of the pits, which were measured along the center line at 10^4^ cycles, as illustrated in Figure 11. In the distribution obtained by using the delta function, each pit had almost uniform width and depth. On the other hand, distributions obtained by using the Gaussian and Weibull distributions exhibited that there were variations in both the width and depth of pits, and the tendency of the distribution obtained by using the Weibull distribution was particularly prominent.

Weibull distribution is based on the weakest-link model to describe the fracture behaviors and is often used as the distribution of the yield stress and static and fatigue strength [44,45]. The impact pressure caused during the collapse of the cavitation bubble depends on the size of the cavitation bubbles, the distance of cavitation bubbles to a solid wall and the pressure fluctuations around the cavitation bubbles. Then, cavitation damage was estimated assuming the impact pressure distribution considering these uncertainties in this study. The plastic deformation occurs when the impact pressure becomes higher than the yield stress, and the plastic deformation area expands as the impact pressure increases. As for the growth behavior of the cavitation damage, the plastic deformation, the crack from the tip of the pit and the detachment of grains generate in order. In other words, it can be said that a localized fatigue phenomenon occurs due to repeated impact pressure loading. Therefore, distributions of the accumulated plastic strain estimated by using the Weibull distribution reproduced the depth profile of the pit better than those using the delta function and the Gaussian distribution, and it is considered that the application of the Weibull distribution is preferable to predict the cavitation erosion phenomenon.

## 6. Conclusions

Cavitation damage on a mercury target vessel for pulsed spallation neutron sources that is induced by proton beam injection in mercury is one of the most crucial issues in realizing sufficient durability under high-power operation. The dispersion of the Gaussian distribution of the maximum localized impact pressure due to cavitation bubble collapsing was evaluated inversely using Bayesian optimization. The prediction method of the cavitation damage using Monte Carlo simulations was proposed taking the uncertainties on the core position of cavitation bubbles and localized impact distributions into account. In the Monte Carlo simulations, the probability distribution of the maximum value of localized impact was assumed to be three kinds of distributions: the delta function and Gaussian and Weibull distributions. The following results were obtained:

(1) Regardless of the probability density function type of the maximum localized impact pressure, the expected reference value of the maximum impact pressure and the dispersion of the Gaussian distribution of the maximum impact pressure were 1.2–2.9 GPa and 1.0–1.7 μm, respectively. These values were within the range of the experimental results.
(2) Although no significant differences in the fraction obtained by using probability density functions were found, the two-dimensional distribution of pits and the histogram of the pit diameter obtained by the Weibull distribution sufficiently reproduced the experimental results.
(3) The depth profile of pits estimated by using the Weibull distribution reproduced the experimental results better than that using the delta function and the Gaussian distribution.
(4) As results of (2) and (3), it can be said that the cavitation erosion phenomenon is predictable by adopting the Weibull distribution.

This prediction method is expected to be applied to predict the cavitation damage in fluid equipment such as pumps and fluid parts.

## Data Availability

Not applicable.
